# Bioactive Phytochemicals in Experimental Models of Multiple Sclerosis: Mechanisms, Efficacy, and Translational Potential

**DOI:** 10.3390/nu18020278

**Published:** 2026-01-15

**Authors:** Weimin Guo, Simin Nikbin Meydani, Dayong Wu

**Affiliations:** 1 Department of Pathology and Laboratory Medicine, Boston University Chobanian and Avedisian School of Medicine, 670 Albany Street, Boston, MA 02118, USA; 2 Nutritional Immunology Laboratory, Jean Mayer USDA Human Nutrition Research Center on Aging at Tufts University, 711 Washington Street, Boston, MA 02111, USA; 3 Friedman School of Nutrition Science and Policy, Tufts University, Boston, MA 02111, USA

**Keywords:** multiple sclerosis, experimental autoimmune encephalomyelitis, gut microbiota, green tea, epigallocatechin-3-gallate, curcumin, resveratrol, dietary phytochemicals

## Abstract

Multiple sclerosis (MS) is a chronic autoimmune disorder of the central nervous system marked by inflammatory demyelination and progressive neurodegeneration. Although current immunomodulatory therapies can reduce relapse rates, they are often associated with limited long-term efficacy and adverse effects, highlighting the need for safer and more comprehensive complementary approaches. Dietary bioactive phytochemicals—notably, the polyphenols epigallocatechin-3-gallate (EGCG), curcumin, and resveratrol—have demonstrated potential to modulate the immune and inflammatory pathways implicated in MS pathogenesis. In addition to their immunomodulatory roles, emerging evidence suggests that these compounds also exert neuroprotective effects independent of immune modulation, including antioxidant activity, mitochondrial stabilization, and enhancement of neurotrophic signaling. Furthermore, recent studies identify the gut microbiota as a central mediator of MS pathophysiology and of how dietary phytochemicals are metabolized and exert their effects. This review examines experimental data evaluating the therapeutic potential of selected bioactive phytochemicals in MS, focusing on their mechanisms of action—including both immune-dependent and immune-independent neuroprotective effects—and interactions with the gut microbiota. Current limitations in translating findings from animal models to clinical settings are also discussed, and future directions for research in this evolving area are highlighted.

## 1. Introduction

Multiple sclerosis (MS) is a chronic immune-mediated disorder of the central nervous system (CNS) characterized by focal inflammatory demyelination, axonal/neuronal damage, and gliosis [1,2,3]. MS most commonly presents in early adulthood (ages 20–40) and is a leading cause of non-traumatic disability in this age group. Global burden estimates exceed 2.5 million cases, including more than 400,000 in the United States, and incidence has been increasing in many regions [4,5,6].

While the exact cause of MS remains unknown, it is considered multifactorial. As with several other autoimmune diseases, the current view is that MS likely reflects dysregulated immune responses shaped by interacting genetic, environmental, and behavioral factors. Cases cluster in families, and first-degree relatives of MS patients have a 10–50-fold increased risk compared with the general population [7]. Additionally, a genome-wide association study comparing MS patients and healthy controls identified 48 genetic variants associated with MS risk [8]; MHC class II genes, along with loci encoding other immune-relevant and neurobiological pathway molecules, have been linked to MS susceptibility [9]. However, genetic predisposition contributes only a minority of risk for MS, roughly 25% of the lifetime risk according to current estimates [6], and epidemiological studies implicate multiple environmental contributors to MS susceptibility. Reported associations include viral exposures (for example, Epstein–Barr virus, human endogenous retroviruses, measles/canine-distemper-related agents, and human herpesvirus-6), cigarette smoking, and nutritional or metabolic factors such as vitamin D deficiency, high salt intake, and obesity [10,11,12,13,14,15,16,17,18].

Recent evidence indicates that interactions between genetic and environmental factors influence individual susceptibility to MS. Environmental exposures can modify gene expression through epigenetic mechanisms such as DNA methylation, histone post-translational modifications, and regulation by non-coding RNAs [19]. Altered DNA methylation is associated with the pathogenesis of MS and changes in the T cell profiles in humans [20,21]. The involvement of micro-RNA (miRNA) in MS is also demonstrated across multiple study types, including patient tissue analyses and experimental models [22,23,24]. For example, miR-34a, miR-155, and miR-326 were upregulated in the brains of patients with active MS, which might be responsible for decreased expression of CD47, a protein that suppresses macrophage activity in active lesions [25].

Clinical manifestations of MS are diverse and commonly include fatigue, mood disorders (depression and anxiety), spasticity and motor incoordination, chronic pain, cognitive impairment, visual and speech disturbances, sensory deficits, bladder and bowel dysfunction, sexual dysfunction, and reduced mobility [26,27]. MS remains incurable; current management relies primarily on immunomodulatory drugs developed largely from animal models that only partially replicate human pathogenesis. These therapies either broadly suppress immunity or selectively target specific immune molecules or pathways. Although they have improved disease control, their efficacy—especially in progressive MS—is limited, and they can produce significant adverse effects. Consequently, interest has grown in complementary and adjunctive approaches to enhance treatment outcomes. Dietary modification is particularly attractive because many dietary components can influence immune and inflammatory responses [28,29,30], in particular, those closely involved in the pathogenesis of MS as discussed in detail in the following sections [31,32,33]. Thus, dietary intervention may represent a promising strategy for both preventive and therapeutic fronts of MS management. Based on the research in this area, many bioactive compounds of plant origin stand out as candidates for this purpose given their demonstrated efficacy in influencing immune and inflammatory responses. In this article, three representatives of such dietary phytochemicals were chosen based on the relatively strong evidence reported in the literature to give a critical review regarding their beneficial effect on experimental MS, working mechanisms, potential translational values, and limitations. To better understand how these phytochemicals work to mitigate MS development, the review begins with a description of the immunopathology in MS pathogenesis based on the current view.

## 2. Immune Responses in Pathogenesis of MS

MS pathogenesis involves autoreactive myelin-specific T cells, together with B cells and innate immune cells, orchestrating CNS demyelination [34,35,36,37,38]. Much of the current understanding of MS immunopathology derives from experimental studies in animal models, notably experimental autoimmune encephalomyelitis (EAE), the best-characterized and most reproducible rodent model of human MS. EAE is typically induced in susceptible animals by immunization with whole-brain homogenate or defined myelin antigens such as basic myelin protein (BMP), proteolipid protein (PLP), myelin oligodendrocyte glycoprotein (MOG), or peptides derived from these proteins such as PLP peptides_139–151_ and MOG peptides_35–55_. Although EAE and MS differ in important respects, many CNS pathologies seen in EAE—such as inflammatory demyelination and axonal injury—recapitulate features observed in MS patients [39,40]. EAE is a powerful model that has been instrumental in elucidating disease mechanisms, developing therapies, and evaluating treatment efficacy for MS. PLP_139–151_-induced EAE in SJL/J mice and MOG_35–55_-induced EAE in C57BL/6 mice are two of the most commonly used models, which produce remitting–relapsing and monophasic progressive patterns, respectively. However, no animal model fully reproduces the complete clinical and pathological spectrum of MS.

Both innate and adaptive immunity—and their interactions—drive the initiation and progression of MS/EAE. Within the innate arm, multiple cell types contribute, but macrophages and microglia are central to pathology. Early in disease, microglia/macrophages adopt a pro-inflammatory (M1-like) phenotype, producing cytokines that injure CNS tissue. At later stages, these cells can shift toward an alternatively activated (M2-like) phenotype, secreting anti-inflammatory mediators that promote resolution and tissue repair [41]. Microglia and macrophages can be either protective or pathogenic depending on time and location [42,43]. Detrimental actions include tissue toxicity, release of proteases, inflammatory cytokines, and reactive oxygen species, and promotion of T-cell recruitment and reactivation in the CNS. Beneficial roles include clearance of debris, secretion of neurotrophic factors, and support for axonal regeneration and remyelination, as observed in EAE models [44].

Adaptive immunity comprises humoral (B cell) and cell-mediated (T cell) responses. T cells are broadly divided into CD4+ helper (Th) and CD8+ cytotoxic (Tc) subsets, with Th cells further subdivided by their characteristic cytokine profiles. CD4+ T cells are considered primary drivers in EAE: in this model, myelin antigens are processed by antigen-presenting cells—principally dendritic cells—which present peptide–MHC complexes that activate autoreactive T cells [45]. Peptide–MHC complexes are presented to CD4+ T cells in peripheral lymphoid tissues (for example, lymph nodes), driving activation, clonal expansion, and differentiation into effector Th subtypes. These pathogenic T cells then cross the blood–brain barrier and enter the CNS, where they release cytokines, activate innate immune cells, and can be reactivated by local antigen presentation (by dendritic cells, microglia, or macrophages), thereby amplifying inflammatory cascades that cause tissue damage. It should be noted, however, that this framework is largely derived from animal EAE models in which defined autoantigens reproducibly induce disease; by contrast, identification of clear target T-cell antigens in human MS has proven much more difficult.

The early study on immuno-pathogenesis of MS was mainly guided in the context of Th1/Th2 paradigm. Th1 cells are defined as a pro-inflammatory phenotype while Th2 cells are believed to be an anti-inflammatory type [46,47]. A disturbed Th1/Th2 balance was the mainstream theory to explain the pathogenesis of MS [48]. MS was long considered a Th1-mediated disease, based on the presence of hallmark Th1 features in lesions—elevated IFN-γ levels and activated macrophages. However, the studies in the past decade have substantially reshaped the landscape of MS immunopathology. In addition to Th1, compelling evidence now implicates IL-17-producing Th17 cells as key contributors to MS/EAE and other T cell-mediated autoimmune diseases [49,50,51]. EAE can be induced by adoptive transfer of either Th17 or Th1 cells, but the two subsets produce distinct clinical phenotypes and pathological features [52], cell infiltration and corresponding chemokine expression [53]. Th17 cells release several pro-inflammatory cytokines, including IL-17A, which downregulates tight junction proteins in BBB, thus allowing the migration of both soluble inflammatory molecules and other circulating immune cells into the CNS [54]. Progress continues to be made as the later studies have identified several distinct populations of CD4+ T cells that are involved in MS/EAE pathogenesis, such as IL-9-producing Th9 cells [55,56], as well as the more recently described Th22 subset, which secretes a distinct effector cytokine profile, including IL-22, IL-13, and TNF-α [57].

Regulatory T (Treg) cells (CD4+CD25+Foxp3+) constitute a specialized effector CD4+ subset that counterbalances pro-inflammatory T cell responses and is protective in MS/EAE. Their primary function is to maintain self-tolerance and restrain excessive immune activation induced by pathogens or environmental triggers, and they have a pivotal role in MS pathogenesis [58]. Reduced Treg numbers and/or impaired suppressive function have been reported in MS patients compared with healthy controls [59,60,61,62,63,64]. In EAE models, disease severity increases after Treg depletion and is ameliorated by the adoptive transfer of Treg cells [65,66].

CD8+ T cells recognize antigens presented on MHC-I, which is expressed by all nucleated cells, whereas CD4+ T cells respond to antigens on MHC-II displayed by antigen-presenting cells. The role of CD8+ T cells in MS/EAE pathogenesis remains incompletely defined [6]. B cells, as professional antigen-presenting cells, efficiently capture antigens via their surface receptors, process them, and present peptide–MHC-II complexes to CD4+ T cells. In MS, B cells can produce myelin-specific antibodies, but their roles in antigen presentation, co-stimulation, and cytokine secretion also contribute importantly to disease pathogenesis [67,68].

In addition to the immune cells mentioned above, some non-immune cells have their roles in MS/EAE pathogenesis. Astrocytes, the most abundant CNS cell type, are essential for maintaining neural homeostasis. In MS lesions, resting astrocytes become reactive and may exert both pro- and anti-inflammatory effects. In addition to supporting blood–brain barrier integrity, astrocytes secrete cytokines and can present antigens to T cells, thereby influencing local immune responses [69,70].

## 3. Neuroprotective Mechanisms Beyond Immunomodulation

While immunomodulation is a key mechanism of phytochemical action in MS, growing evidence indicates that many plant-derived compounds also provide direct neuroprotective effects independent of immune modulation. These actions are especially relevant in progressive MS, where neurodegeneration can proceed with limited overt inflammation and where conventional immunotherapies are often less effective.

Oxidative stress is a major driver of axonal injury and demyelination in MS. Phytochemicals, such as epigallocatechin-3-gallate (EGCG), curcumin, and resveratrol, exhibit antioxidant effects either by directly scavenging reactive oxygen species or by activating endogenous pathways (for example, the Nrf2/ARE axis). EGCG, for example, reduces oxidative damage and lipid peroxidation in neuronal cells and has been reported to protect selectively against mitochondrial oxidative stress [71]. Curcumin enhances endogenous antioxidant defenses and activates the Nrf2/HO-1 pathway in EAE models, contributing to reduced oxidative damage, enhanced myelin repair, and improved neurological function [72,73]. Resveratrol, delivered via nanoparticle formulations, selectively mitigated retinal ganglion cell (RGC) loss in EAE models despite not altering inflammation or demyelination, suggesting antioxidant-mediated neuroprotection [74]. Moreover, resveratrol also contributes to blood–brain barrier (BBB) stabilization via antioxidant and tight junction maintenance mechanisms in EAE [75].

In terms of mitochondrial protection and bioenergetics, these phytochemicals can stabilize mitochondrial function and boost energy metabolism in neurons and glia. Among the phytochemicals examined, EGCG accumulates in mitochondria and exerts selective anti-apoptotic effects against mitochondrial oxidative stress. In primary rat cerebellar granule neuron cultures, radiolabeled EGCG was found predominantly in the mitochondrial fraction (≈90–95%), and EGCG selectively protected neurons from apoptosis induced by mitochondrial oxidative insults but not from other apoptotic stimuli [71]. This targeted mitochondrial action distinguishes EGCG from other non-specific antioxidants and underscores its therapeutic potential in neurodegenerative contexts where mitochondrial dysfunction drives pathology—such as in progressive MS. Curcumin helps maintain mitochondrial membrane potential and reduces mitochondrial permeability transition, protecting neural cells from energy failure and death [76]. Resveratrol further supports mitochondrial integrity by activating SIRT1 and PGC-1α, promoting mitochondrial biogenesis and improving metabolic flexibility under conditions of stress [77,78].

The modulation of neurotrophic and synaptic pathways is another mechanism through which phytochemicals confer neuroprotection. Resveratrol has been reported to upregulate brain-derived neurotrophic factor (BDNF), a key regulator of neuronal survival, synaptic plasticity, and remyelination [79,80,81]. Curcumin similarly activates BDNF signaling via CREB modulation and enhances cognitive and synaptic functions in MS animal models [82,83]. EGCG has also been demonstrated to preserve synaptic structures by reducing glutamate-excitotoxic synaptic loss and safeguarding synaptic protein expression under oxidative stress conditions [84,85].

Finally, reduction in protein aggregation and enhancement of autophagic support represent additional neuroprotective mechanisms. Impaired autophagy and the accumulation of damaged proteins are linked to neurodegeneration in MS. Curcumin restores autophagy impaired in EAE mice by modulating the AKT/mTOR pathway, an effect associated with improved neuronal survival [86]. Meanwhile, resveratrol promotes remyelination and motor recovery in cuprizone models through SIRT1/FoxO1-dependent autophagic activation [87]. EGCG has also been implicated in autophagy regulation, potentially via AMPK activation, although further studies are needed to confirm this effect in MS models [88].

Together, these findings reinforce the multifaceted neuroprotective potential of EGCG, curcumin, and resveratrol. Beyond their well-characterized immunomodulatory properties, these compounds engage a range of cellular pathways that support neuronal health and resilience. By acting on processes implicated in neurodegeneration—particularly relevant in progressive forms of MS—they offer therapeutic promise that extends beyond inflammation control.

## 4. Effects of Bioactive Phytochemicals on MS/EAE and the Underlying Mechanisms Involved

### 4.1. Epigallocatechin-3-Gallate

#### 4.1.1. Overview

Catechins, phytochemicals present in many fruits and tea leaves, are noted for a range of health benefits [89,90]. Epigallocatechin-3-gallate (EGCG), which comprises roughly 50–80% of total catechins, is the most extensively studied and is generally regarded as the most bioactive. EGCG has potent antioxidant, anti-inflammatory, and disease-modulating activities, but its therapeutic use is limited by poor oral bioavailability, instability, and rapid degradation [91,92,93]. Most orally ingested EGCG reaches the colon and is microbially converted into low-molecular-weight metabolites (e.g., phenyl-γ-valerolactones, phenolic acids) with distinct bioavailability and activity, so interindividual microbiota differences strongly affect systemic exposure. In CNS autoimmunity models, EGCG can reduce inflammatory cytokines and modulate T cell responses (dampening Th1/Th17, promoting Tregs), but effects are timing- and tissue-dependent and have been variable across studies [94,95,96,97,98,99]. Preclinical EAE studies consistently show that EGCG reduces disease severity, neuroinflammation, and demyelination while exerting antioxidant and immunomodulatory effects (shifting away from Th1/Th17 responses). Clinical evidence on MS is limited and inconclusive: small trials report acceptable short-term safety and occasional symptomatic benefits, but no robust disease-modifying efficacy has been established [97,99,100,101]. Dietary feasibility must be evaluated case-by-case, since effective concentrations depend on dose, route, regimen, formulation, and individual factors (e.g., gut microbiota), and many preclinical studies do not report systemic EGCG levels. It is notable that EGCG Cmax varies with dose, formulation, feeding state, and species. In humans, Chow et al. (2003) reported a free EGCG Cmax of 0.51 ± 0.31 µM (234.9 ± 140.9 ng/mL, mean ± SD) after a single 800 mg oral dose, rising to 0.85 ± 0.51 µM (390.3 ± 231.4 ng/mL) after 4 weeks of daily 800 mg administration [102]. Ullmann et al. (2003) reported an average Cmax of 2.33 µM (range 1.14–3.10 µM; 1067.38 ng/mL, range 521.86–1417.95 ng/mL) after an 800 mg oral dose, and 7.41 µM (range 2.99–10.32 µM; 3391.60 ng/mL, range 1370.12–4726.11 ng/mL) after a 1600 mg dose; in this study, EGCG capsules were taken under fasting conditions and subjects remained fasting for 2 h post-administration [103]. Henning et al. (2005) similarly measured plasma EGCG concentrations following oral administration of 580 mg purified EGCG taken 20 min after a light breakfast; the reported Cmax was approximately 0.7 ± 0.4 µM (mean ± SE) [104]. In experimental animals, Lambert et al. reported a Cmax of 0.28 ± 0.08 µmol/L (mean ± SD) following intragastric administration of EGCG at 163.8 µmol/kg in male CF-1 mice [105], while Dube et al. reported a Cmax of total EGCG of 0.0343 ± 0.0020 µM (mean ± SD) in Swiss outbred mice orally administered 0.76 mg/kg EGCG [106].

#### 4.1.2. Experimental Results

EGCG was first reported to alleviate EAE by Aktas et al. [45]. In that study, EGCG was delivered by oral gavage to female SJL/J mice (300 µg per mouse, twice daily) from the day of immunization with PLP_139–151_ to induce EAE. EGCG treatment improved clinical scores, reduced brain inflammation and neuronal damage, and was associated with decreased ex vivo T cell proliferation induced by PLP peptide_139–151_ rechallenge and lower TNF-α production stimulated by PMA plus ionomycin stimulation. It is noted that this study preceded later refinements in the characterization of pathogenic T cell subsets in MS/EAE; contemporary studies would typically include additional subset-specific markers to further define EGCG’s immunomodulatory effects. Consistent with these findings, a subsequent study reported [107] that dietary supplementation with EGCG attenuated EAE in a dose-dependent manner. Female C57BL/6 mice were fed AIN-93M diets containing 0, 0.15, 0.3, or 0.6% (*w*/*w*) EGCG starting 30 days before immunization with MOG_35–55_; mice receiving higher EGCG concentrations showed progressively reduced clinical scores, inflammation, and demyelination (the original report did not convert % *w*/*w* to estimated daily intake in mg/kg/day). Clinical improvement was associated with reduced CNS infiltration by CD4+ T cells and, to a lesser extent, neutrophils and macrophages, along with suppressed expansion and effector function of autoreactive T cells after MOG rechallenge (reduced ex vivo T-cell proliferation, attenuated delayed-type hypersensitivity responses, and lower production of pro-inflammatory cytokines, including IFN-γ, IL-17, IL-23, IL-6, TNF-α, and IL-1β). EGCG-fed mice also showed decreased frequencies of Th1 and Th17 cells and lower expression of the master regulators T-bet and RORγt, together with an increased proportion of Treg cells in lymph nodes, spleen, and CNS compared with controls. In agreement but using a different study protocol, Sun et al. [108] later reported that daily intraperitoneal injections of EGCG (300 µg) begun at symptom onset in MOG_35–55_-induced EAE (male C57BL/6 mice) were protective. This effect was associated with the suppression of Th1/Th17 responses and their STAT signaling pathways, and with impaired APC function, as evidenced by the reduced expression of the costimulatory molecules CD80 and CD86 and diminished T-cell stimulatory capacity [108].

EGCG has also been tested as a complementary remedy to synergize the therapeutic efficacy of conventional drugs on MS using mouse models for both the relapsing–remitting form (PLP-induced) [109] and the progressive form (MOG-induced) [110] of MS, in the two studies reported by the same group that published the first study on the protective effect of EGCG in EAE [45]. In one study [109], EGCG was given by oral gavage to SJL/J mice (300 µg per mouse, twice daily), beginning 9 days before immunization with PLP_139–151_, combined with a suboptimal dose of the anti-MS drug glatiramer acetate (GA; 50 µg), administered beginning two days after EGCG; the treatment produced effects not seen with GA alone at this dose: the combination significantly delayed EAE onset, reduced disease severity, and diminished neuroinflammation [109]. In the next study to determine whether combined GA and EGCG therapy had a synergistic effect in progressive MS, the authors immunized C57BL/6 mice with MOG_35–55_ to induce EAE, and the same EGCG (300 µg, twice daily) dose with suboptimal doses of GA (50 µg/d for prevention, and 150 µg/d for treatment) was used [110]. Results showed that EGCG alone, and even GA alone, but to a lesser degree, had a protective effect. However, unexpectedly, combination therapy had no effects, and it appeared that GA abolished the protective effect of EGCG’s neuroprotective effect. Consistent with this, both GA and EGCG monotherapies reduced spinal-cord inflammation, whereas the GA+EGCG combination did not produce an additional effect; likewise, no differences were observed between the EGCG alone and EGCG+GA groups in ex vivo proliferation of pathogenic T cells or in the proportions of Th1, Th17, and Treg cells among CNS-infiltrating CD4+ T cells. Interestingly, EGCG reduced axonal damage and demyelination whereas GA did not; when used together, the neuroprotective effect of EGCG was absent. These results may partly explain the abolished beneficial effect of EGCG when co-administered with GA, although future studies are needed for further elucidation.

There are only two reported clinical trials with the use of EGCG in MS patients. The first study involved eighteen patients with relapsing–remitting MS and employed a randomized, double-blind, placebo-controlled crossover design. Participants received 600 mg/day of EGCG and placebo each for 12 weeks, with a 4-week washout period between treatments. [111]. However, the authors reported that EGCG improved muscle metabolism during moderate exercise but did not assess neurological or immunological outcomes. Another study comprised a combined phase I (single-arm) and phase II (randomized, placebo-controlled) trial evaluating the safety and potential neuroprotective effects of “Polyphenon E,” a green-tea extract standardized to ~50% EGCG [112]. In this study, MS patients received two Polyphenon E capsules (200 mg EGCG per capsule), twice daily (total 800 mg EGCG/d) for six months. In the phase I portion, 10 participants completed the study with no serious adverse events; one participant withdrew due to grade-1 liver-function abnormalities. In the phase II randomized trial, 13 participants were enrolled and 12 began treatment, but the study was terminated prematurely after an unexpectedly high rate of elevated liver enzymes (five of seven) was observed in the Polyphenon E group. The signal was temporally associated with a new production batch of Polyphenon E, but quality-control testing found similar EGCG concentrations in both lots, and the reason for the differing toxicity remained unclear. Consequently, these trials do not confirm or refute the protective effects seen in EAE models, and the clinical benefit of EGCG in MS remains unresolved. Table 1 summarizes the effects of EGCG in preclinical EAE studies.

#### 4.1.3. Mechanisms

The protective effect of EGCG on MS/EAE is believed to be mainly mediated through its immuno-modulating effect, although its antioxidant property may also help prevent tissue damage and oxidative stress—and its interplay with inflammation—contributes substantially to neuronal injury in MS/EAE [113]. Because T cell-mediated autoimmunity drives MS/EAE, both the broad suppression of T cell responses and the selective modulation of specific T cell subsets and signaling pathways can yield therapeutic benefit to varying degrees. EGCG affects multiple facets of innate and adaptive immunity [96]. Since MS/EAE is primarily T cell-mediated, the following discussion focuses on EGCG’s effects on T cell biology. Studies indicate that EGCG influences multiple T cell processes, including activation, proliferation, differentiation, and effector functions such as cytokine production and cross-talk with innate immune cells.

Several studies have shown that in vitro EGCG or green tea extract supplementation inhibit T cell proliferation [114,115,116]. The suppression of T-cell proliferation by EGCG may limit the activation and expansion of autoreactive T cells, reducing CNS inflammation, pro-inflammatory cytokines (notably Th1/Th17), and promoting regulatory T-cell responses in animal models. Reported mechanisms include interference with T-cell signaling (for example, IL-2/IL-2R) and cell-cycle progression. However, in vitro proliferation assays do not capture in vivo complexity—effects on trafficking, subset differentiation, innate and CNS-resident cells, and achievable systemic concentrations must also be considered—and clinical evidence of disease-modifying benefit in humans remains limited. Furthermore, since the concentrations (50–200 µM) used in these studies were much higher than the peak blood concentrations ever reported after oral consumption (<10 µM), the physiological relevance of these results are not clear. To address this issue, Wu et al. tested physiologically relevant EGCG concentrations (2.5–10 µM) and found that EGCG dose-dependently inhibited T cell proliferation and cell cycle progression. They further showed that this antiproliferative effect was independent of oxidative stress, cytotoxicity, or apoptosis [117]. In a subsequent study [118], these authors also reported that EGCG’s suppressive effect was greater on CD4+ than on CD8+ T cells. In an antigen-specific model, EGCG reduced T-cell proliferation by acting on both T cells and antigen-presenting cells, with a predominant direct effect on T cells. Furthermore, they confirmed the T cell-suppressive effect of EGCG under the in vivo condition by showing that feeding mice 0.3% EGCG for 6 weeks was effective in reducing ex vivo T cell proliferation, suggesting that dietary EGCG supplementation could achieve in vivo concentrations sufficient to suppress T cells.

EGCG’s effects on T-cell responses appear to involve T-cell receptor (TCR)-associated signaling, downstream pathways, gene transcription, and cytokine production. In Jurkat T cells, EGCG inhibited early activation events (ZAP-70, LAT, PLCγ1), suppressed MAPK activity, and reduced AP-1 transcriptional activation [119]. Wang et al. [120] found that EGCG did not alter IL-2 production but reduced expression of all three IL-2R subunits (IL-2Rα, IL-2Rβ/CD122, and the common γ chain/CD132). This downregulation impaired IL-2R signaling, as shown by decreased STAT5 phosphorylation in naïve CD4+ T cells treated with EGCG.

Evidence indicates that the dysregulation of specific CD4+ T-cell subsets, rather than a generalized hyperresponsiveness, is a critical pathogenic factor in MS/EAE [6,121]. EGCG’s benefits in EAE align with shifts in CD4+ T-cell subsets—reduced Th1 and Th17 frequencies and increased Treg proportions—accompanied by corresponding changes in their master regulators [107,108]. These results suggest that EGCG modulates CD4+ T-cell differentiation. Supporting this, an in vitro study showed that EGCG inhibited the differentiation of naïve CD4+ T cells into Th1, Th9, and Th17 subsets by interfering with their respective regulatory networks. Although EGCG did not directly promote Treg differentiation in that study, it shifted the Th17–Treg balance in favor of Treg by inhibiting IL-6 signaling [122].

In EAE, peripherally induced autoreactive T cells must cross the blood–brain barrier (BBB) to initiate CNS disease. Reduced CNS infiltration of pathogenic T cells in EGCG-treated mice may reflect improved BBB integrity, as suggested by Wang et al. [107]. In that study, EGCG supplementation lowered the plasma levels of soluble intercellular adhesion molecule-1 (sICAM-1) and reduced the number of CD4+ T cells expressing the chemokine receptor CCR6 in EAE mice. Adhesion molecules and chemokine receptors regulate immune-cell migration across the blood–brain barrier, and elevated serum sICAM-1 has been reported in MS patients [123,124]. Animal EAE studies have demonstrated that ICAM-1 promotes CNS infiltration by pathological cells and contributes to disease development [125,126]. CCR6 facilitates entry of the initial wave of autoreactive Th17 cells into the uninflamed CNS by binding its sole ligand, CCL20, which is constitutively expressed by choroid plexus epithelial cells [127]. These findings may help to explain the EGCG-induced reduction in T-cell infiltration; however, CCL20 is unlikely to mediate this effect, since EGCG did not alter CCL20 expression in the CNS of EAE mice [107]. Together, these findings point to an additional mechanism that may contribute to EGCG’s beneficial effects in EAE.

In summary, the evidence indicates a dynamic interplay between green-tea catechins and the gut microbiota: catechins alter microbial composition and function, microbes convert catechins into bioactive metabolites, and gut dysbiosis appears to contribute mechanistically to MS/EAE. These converging lines of evidence support the proposition that green tea/green tea catechins may influence MS/EAE not only via direct immune modulation but also indirectly via shaping the gut-microbiota–metabolite axis. Future studies should identify the specific microbial taxa and metabolites mediating these effects, quantify interindividual variability in microbial responses to catechins, and evaluate targeted dietary or probiotic strategies to optimize the microbiota–metabolite axis for the prevention or amelioration of MS/EAE.

### 4.2. Curcumin

#### 4.2.1. Overview

Curcumin, a hydrophobic polyphenol that constitutes about 2–5% of the spice turmeric, exhibits anti-inflammatory and antioxidant activities and has shown protective effects in autoimmune and inflammatory conditions such as rheumatoid arthritis, type 1 diabetes, colitis, and MS. These actions likely reflect curcumin’s modulation of both innate and adaptive immunity. Curcumin can target innate immune receptors (for example, Toll-like receptors), intracellular signaling pathways (MAPK, STAT), and transcription factors (NF-κB, AP-1) to suppress the production of pro-inflammatory cytokines, prostaglandin E2, reactive oxygen and nitrogen species, and chemotactic signals. It generally inhibits T-cell proliferation and cytokine production, with differential effects across T-cell subsets, and can impair dendritic cell maturation, suggesting an additional route to modulate antigen-specific T-cell responses. These immunomodulatory properties have prompted the testing of curcumin in EAE models. Interest has also grown in curcumin’s interactions with the gut microbiome: emerging evidence indicates that curcumin supplementation can alter microbial composition and function, potentially influencing the microbiota–gut–brain axis and contributing to its immunomodulatory and neuroprotective effects [128,129,130,131,132]. Curcumin might improve MS at least partially through modulating gut microbiota [133].

#### 4.2.2. Experimental Results

Several studies have demonstrated curcumin’s efficacy in EAE models. In a study using both EAE models, induced by active immunization (with mouse spinal cord homogenates) or by adoptive transfer (cells from donor mice immunized with MBP), curcumin administration (intraperitoneal injection of 50 or 100 µg/d on every other day from 0 to 25 days) started at the same time of induction reduced the duration and clinical severity of EAE in SJL/J mice, which is associated with decreased IL-12 production by macrophages/microglia and the reduced differentiation of myelin-specific Th1 cells [134]. Using the in vitro model to determine the working mechanisms, the authors found that curcumin-induced inhibition of the Janus kinase-STAT pathway mediated the decrease in IL-12-induced T cell proliferation and Th1 differentiation [134,135]. The same group later reported a similar protective effect of curcumin in different EAE models, i.e., SJL/J and C57BL/6 mice immunized with PLPp_139–151_ and MOGp_35–55_, respectively; furthermore, ameliorated EAE disease scores were accompanied by the decreased expression of TLR4 and TLR9 in both CD4^+^ and CD8^+^ T cells [136]. In a follow-up study, they showed that curcumin-treated EAE mice produced lower levels of IFN-γ, IL-17, IL-12, and IL-23 [135], which is consistent with results from an MBP-induced rat EAE model, where oral curcumin (100 or 200 mg/kg) reduced disease severity and inflammatory infiltration and lowered spinal-cord mRNA levels of IL-17, TGF-β, IL-6, IL-21, STAT3, and RORγt [137]. In a more recent study [138], Bruck et al. not only confirmed the protective effect of curcumin in EAE but also further demonstrated that curcumin inhibited Th1 and Th17 response but promoted Th2 response; moreover, curcumin induced an anti-inflammatory phenotype in DC and suppressed IL-12p40 and IL-23p19 expression. Interestingly, curcumin had no effect on ovalbumin-induced airway inflammation, suggesting the specificity of curcumin in affecting organ-specific inflammatory disorders. In one study, curcumin monoglucuronide (CMG), a curcumin prodrug that yields high serum levels of free curcumin after injection, reduced EAE severity both clinically and histologically and altered microbiota composition in feces and ileal contents but not in the ileal mucosa [133]. Changes in ileal microbiota composition correlated with clinical and histological outcomes, and specific taxa (*Ruminococcus bromii*, *Blautia gnavus*, *Turicibacter* sp., *Alistipes finegoldii*, *Burkholderia* spp., and *Azoarcus* spp.) were associated with EAE modulation. These results support the idea that CMG differentially reshapes gut microbiota across intestinal sites and that such site-specific shifts are linked to the suppression of CNS inflammation. However, the causality and the precise immune pathways mediating microbiota-to-CNS effects were not established and warrant further mechanistic study. Table 2 summarizes the effects of curcumin in preclinical EAE studies.

#### 4.2.3. Mechanisms

Similarly to EGCG, as a phenolic phytochemical with anti-inflammatory, immuno-modulating, and antioxidant properties, curcumin’s protective effect on EAE is primarily mediated by its desirable influence on immune cell functions as well as attenuating the tissue damage caused by inflammatory and oxidative insults.

Phagocytes—mainly neutrophils and monocytes/macrophages—are the first responders of the innate immune system to infection. They eliminate pathogens via reactive oxygen and nitrogen species, proteolytic enzymes, antimicrobial peptides, and by recruiting additional immune cells. When activated instead by autoreactive T cells, as in MS/EAE, these same mechanisms can damage host tissue. Curcumin reduces phagocyte recruitment and activity under various conditions; for example, it inhibits neutrophil and monocyte/macrophage migration by suppressing chemokines (IL-8, MCP-1) and downregulating adhesion molecules (ICAM-1 and VCAM-1) [139,140,141]. Multiple studies have shown that curcumin suppresses monocyte/macrophage production of inflammatory mediators and reactive oxygen/nitrogen species (see review [142]). Microglia are considered the primary cell type directly responsible for tissue damage in MS/EAE. However, the studies cited above assess curcumin’s anti-inflammatory effects in broad contexts rather than specifically within MS/EAE, making it difficult to distinguish whether curcumin acts by directly suppressing innate immune cell activity or indirectly by reducing activation signals from CD4+ T cells. Given the available evidence, it is reasonable to speculate that both direct and indirect mechanisms contribute.

Curcumin has been shown to broadly suppress T cell functions across diverse experimental settings including both in vivo and in vitro using different T cell activation agents, and also more relevantly, in EAE models. An in vitro study using mouse spleen cells showed that at 6.25–30 μM, curcumin inhibited mitogen- and antigen-induced lymphocyte proliferation, T cell cytotoxicity, IL-2 and IFN-γ production, and NF-κB activation [143]. Similar results were also observed in the studies using human PBMC [144], spleen cells [145], and purified blood T cells [146]. The studies have expanded to examine the effect of curcumin on specific T cell subpopulations known to play key roles in pathogenesis of MS/EAE. In an in vitro study using PBMC from both healthy individuals and MS patients, Fahey et al. found that at 20 μg/mL, curcumin inhibited IL-12-induced STAT4 phosphorylation, IFN-γ production, and IL-12 receptor expression, while enhancing IFN-β-induced STAT4 phosphorylation and production of the anti-inflammatory cytokine IL-10 [147]. Because IL-12 signaling drives Th1 polarization—mediated by STAT4 and characterized by IFN-γ production—these results imply that curcumin’s suppression of Th1 responses and upregulation of IL-10 may underlie its protective effects in MS/EAE. This is further supported by EAE studies showing that curcumin’s beneficial effects are linked to reduced IL-12 production by macrophages/microglia and decreased differentiation of neural antigen-specific Th1 cells [134]. Th17 cells, a later-defined CD4^+^ T cell subset thought to be a paramount player in MS/EAE development, are shown to be inhibited by curcumin in both the in vitro differentiation experiments and in vivo EAE model analysis as mentioned above [135,137,138]. The effect of curcumin on Treg cells in MS/EAE is relatively less known. An animal study reported that the inhibition of EAE by curcumin was associated with the upregulation of IL-10, PPAR-γ, and Treg cells in the CNS and lymphoid organs [135]. However, given that curcumin is known to enhance Treg and Th2 response in several other autoimmune diseases such as systemic lupus erythematosus in humans [148], and experimental autoimmune myasthenia gravis in animals [149], it appears promising to see more evidence coming out in this regard.

Since MS/EAE are antigen-specific autoimmune processes, self-antigens are recognized by T cells only after being processed and presented by APC, and this makes APC a potential target for curcumin to mitigate MS/EAE development. It seems to be the case based on the study findings in this regard. In vitro studies showed that curcumin (20–25 µM) inhibited DC maturation as indicated by the reduced expression of maturation markers CD83, CD80, CD86, CD40, and MHC-II, production of cytokines IL-12, IL-10, TNF-α, and IFN-γ, and DC-induced T cell proliferation [150,151]. Furthermore, infusion of mice with allogeneic curcumin-treated DC enhanced the development of FoxP3^+^ Treg cells and reduced subsequent alloproliferative capacity [151]. In an antigen-specific reaction system, after curcumin-pretreated DC, OVA peptide-load DC was incubated with OVA-specific TCR-transgenic (OT-II) T cells, and the production of IFN-γ, IL-17, and IL-2 from these T cells were reduced, whereas IL-4 was enhanced [138]. Taken together, curcumin’s protective effect in MS/EAE is mediated via its regulation of both innate and adaptive immune cell functions and APC function that bridges innate and adaptive immunity.

### 4.3. Resveratrol

#### 4.3.1. Overview

Resveratrol, a polyphenolic compound found in at least more than 70 plant species, is particularly enriched in fresh grape skin, which contributes to high concentrations of resveratrol in red wine and grape juice. Epidemiological studies demonstrated that red wine consumption is inversely associated with the incidence of chronic diseases, in particular cardiovascular disease, and resveratrol might play a role in this phenomenon. Indeed, resveratrol has been shown to have a number of biological properties that can be linked to the proposed health benefits based on animal and human studies as well as mechanistic investigation at the cellular and molecular levels [152]. Resveratrol has a strong anti-inflammatory effect in studies using in vitro cell-based experiments, in vivo animal models, and human clinical trials; a key mechanism for its anti-inflammatory activity is mediated by the suppression of NFκB activation and other related pathways (for more detail, see the review in [153]). Given its anti-inflammatory property and its neuroprotective effects, resveratrol has been tested in EAE animal models. Preliminary results appeared promising as discussed in more detail in the following sections. Gut microbiota dysbiosis plays a key role in the pathogenesis of MS/EAE, and the beneficial effects of resveratrol may be, at least partially, mediated through its capacity to modulate gut microbial composition, preserve intestinal barrier integrity, and attenuate intestinal inflammation [154,155]. However, direct evidence connecting the resveratrol-induced modulation of gut microbiota with improvements in dysbiosis and disease outcomes in MS/EAE remains limited. Future studies should focus on elucidating the causal links between resveratrol-mediated microbial changes and neuroinflammatory modulation, integrating multi-omics approaches to define key microbial taxa, metabolites, and host pathways involved in its gut–brain axis effects.

#### 4.3.2. Experimental Results

The first evidence for the protective effect of resveratrol in EAE was published only 10 years ago, almost simultaneously, by two groups [156,157]. In the study, by using a MOG_35–55_-induced EAE animal model in C57BL/6 mice (monophasic progressive model), Singh et al. demonstrated that oral gavage of resveratrol starting right after immunization decreased clinical symptoms and inflammation in the spinal cord, and decreased serum levels of pro-inflammatory cytokines and chemokines, including TNF-α, IFN-γ, IL-2, IL-9, IL-12, IL-17, MIP-1α, MCP-1, RANTES, and Eotaxin [157]. In the other study, PLP_139–151_ peptide was used to induce the EAE model in SJL/J mice, a remitting–relapsing EAE model. However, the main purpose of using this EAE model in that study was to produce optic neuritis, an inflammatory demyelinating disease of the optic nerve, and then determine whether resveratrol had protective effects. At 0, 3, 7, and 12 days post-immunization, SRT501, a pharmaceutical-grade formulation of resveratrol, was administered via intravitreal injection, and it was found that it significantly attenuated retinal ganglion cell loss during acute optic neuritis but had no effect on optic nerve inflammation. However, no effect of SRT501 on EAE symptoms (tail and limb paralysis) was found, which is not surprising because it was administered locally, and thus, no substantial levels would be expected beyond the site. Thus, the role of resveratrol in general MS was not really addressed in this study. As a follow-up, the same group later conducted another study using the same EAE model in which SRT501 was systemically administered by oral gavage [158]. They showed that SRT501 treatment attenuated neuronal damage in both optic nerves and spinal cords but had no effect on EAE onset and symptom scores. Although SRT501 treatment reduced EAE symptoms during the remission on days 20–25, it did not prevent the onset of EAE remission on day 30. Interestingly, SRT501 treatment affected neither gross inflammation nor the phenotype of inflammatory cell infiltrates in the spinal cord at the peak of EAE [158]. Next, to determine whether these findings obtained in the remitting–relapsing EAE model were also true of the progressive EAE, as well as for comparison between the purified form and the modified form of resveratrol, Shindler’s group showed that both resveratrol and SRT501, when orally administered to MOG_35–55_-induced EAE in C57BL/6 mice, delayed the onset of EAE by several days, prevented neuronal loss, and delayed visual decline in EAE mice; however, resveratrol had no effects on inflammation in the spinal cord and optic nerves, the number of T cells in the periphery and CNS, and the percentage of CD4^+^ vs. CD8^+^ T cells present in the spinal cord or spleen in EAE mice [159]. Based on these results, the authors proposed that neuroprotective effect can occur without immunosuppression. In contrast to these results, in another study using the same EAE model, i.e., induced by PLP_139–151_ peptides in SJL/J mice, dietary resveratrol supplementation (0.02, 0.04, 0.08% in diet) was shown to improve EAE symptoms, and also differentially regulated cytokine IL-17, IFN-γ, and TNF-α production by spleen cells after PLP rechallenge [160]. They also found that resveratrol treatment was associated with higher proportions of IL-17^+^IL-10^+^ CD4^+^ T cells and IFN-γ^+^CD4^-^ T cells in the spleen and brain, respectively. Additionally, they showed that resveratrol reduced the severity of EAE induced by the adoptive transfer of encephalitogenic T cells [160]. It should be noted that compared to the study by Shindler et al. [158], this study used the purified form of resveratrol provided in the diet, started the feeding earlier, and lasted longer. These differences in study protocol should be kept in mind when making comparisons to the study results.

Recently, Dr. Wang et al. reported that resveratrol attenuated the severity of clinical EAE, and protected BBB integrity using a MOG_35–55_-induced EAE model in C57BL/6 mice, which was associated with maintained tight junction protein expression and reduced expression of adhesion molecules [75]. This group also reported that resveratrol had a synergistic effect in reducing EAE symptoms, spinal cord inflammation, and serum pro-inflammatory cytokines IFN-γ and TNF-α, as well as increasing serum anti-inflammatory cytokines IL-4 and IL-10 when combined with the intravenous infusion of mouse bone marrow mesenchymal stem cells [161]. In addition to the two commonly used EAE models, Ghaiad et al. reported that resveratrol could attenuate the impairment in balance and locomotor coordination, reverse cuprizone-induced demyelination, improve brain mitochondrial function, and reduce cuprizone-induced oxidative stress and inflammation in a copper chelator cuprizone-induced EAE model [162].

However, the beneficial effects of resveratrol in the EAE model are not consistently reported. For example, Sato et al. [163] reported that resveratrol treatment was not protective, but instead, it even exacerbated the diseases in both EAE and TMEV-IDD models, as indicated by more severe clinical symptoms and pathological changes, by using both a MOG_35–55_ peptide-induced EAE model in C57BL/6 mice, and a Theiler’s murine encephalomyelitis virus-induced demyelinating disease (TMEV-IDD) in SJL/J mice, and resveratrol was administered (0.04% in diet, or 20 mg/kg/d) during the induction (days −1 to 8), effector (days 14 to 23) phase, or through the whole course (days −1 to 63) in the EAE model, and during the chronic phase (days 35 to 48) of TMEV infection in TMEV-IDD model. The reasons for the discrepancy between this study and the others discussed above remain unclear. Given that this is the sole animal study reporting a harmful outcome so far, further studies are required to confirm or refute this finding before any definitive conclusions can be made. Table 3 summarizes the effects of resveratrol in preclinical EAE studies.

#### 4.3.3. Mechanisms

Given that pathogenesis of MS/EAE involves antigen-specific autoimmune response, BBB leakage, immune cell infiltration into CNS, inflammation, oxidative stress, demyelination, axonal/neuronal damage, and gliosis, the protective effects of resveratrol on MS/EAE might be mediated through multiple routes. Like EGCG and curcumin, resveratrol has anti-inflammatory and antioxidant properties, which may contribute to its protective effect demonstrated in the studies described above. Although less information is available relative to the other two phytochemicals, resveratrol seems to also differentially impact different immune cells, rather than having a universal suppression, in achieving a condition that would ameliorate the disease development. For example, resveratrol is shown to inhibit the production of pro-inflammatory cytokines (IFN-γ, TNF-α) and increase the production of anti-inflammatory cytokines (IL-4, IL-10) [161]. In the context of T cell populations, resveratrol-induced improvement in EAE is linked to the increased populations of IL-17^+^IL-10^+^ T cells and CD4^−^IFN-γ^+^ cells in brains and spleens, as well as the repressed expression of IL-6, IL-12/23 p40, IL-12 p35, and IL-23 p19 in macrophages [160], the cytokine phenotypes known to drive pro-inflammatory Th1 and Th 17 differentiation. Unlike EGCG and curcumin, resveratrol was found to improve EAE symptoms and neurological pathology without affecting inflammation, and immune cell phenotype and their functions when it is administered late (after the induction phase) [158,159]. In the studies showing that effects of resveratrol on EAE symptoms and pathology were accompanied by altered inflammation and immune cell activities, resveratrol administration started earlier, usually right after immunization. It is possible that when resveratrol was given after the autoimmune process was fully developed, resveratrol was incapable of reversing the immune and inflammatory events; however, it is still effective in preventing and reducing the subsequent neurological damages induced by inflammatory and oxidative attacks; additionally, studies have suggested that resveratrol may promote the repair and recovery of damaged tissues in the CNS [164,165]. In contrast, in the study by Singh et al., resveratrol treatment started right after immunization and authors observed not only improved clinical symptoms and pathology in the spinal cord but also a reduction in both local (spinal cord) and systemic inflammation (serum pro-inflammatory cytokines and chemokines) [157]. Similarly, in the above-mentioned study by Imler et al. [160], dietary resveratrol supplementation in PLP_139–151_ peptide-induced EAE in SJL/J mice also resulted in some desirable changes in the immune cell phenotype and cytokine production in addition to improved EAE symptoms. Thus, resveratrol indeed has an immunomodulatory effect, and this effect contributes to its protective role in EAE.

In addition to its effect on immune and inflammatory responses, resveratrol has been found to affect the functions of non-immune cells involved in MS/EAE, which may also contribute to its protective effect. In MS/EAE, autoreactive immune cells induce impaired integrity of BBB to gain entry into the CNS. In the study mentioned above [75], resveratrol-induced improvement in EAE was associated with the increased integrity of BBB, which may be mediated through preventing EAE-induced loss of tight junction proteins, including ZO-1, occludin, claudin-5, and inhibiting the upregulation of adhesion molecules (ICAM-1 and VCAM-1) in the brain. These results are consistent with the observed effect of resveratrol improving impaired BBB integrity in several other animal models such as stroke (ischemia) [166], and high fat-induced obesity [167].

Resveratrol is an activator of SIRT-1, a mammalian NAD-dependent deacetylase [168]. SIRT-1 has been demonstrated to have several health benefits by playing a role in regulating a variety of cell functions, including protective effects in neurodegenerative diseases. Therefore, resveratrol may function as a SIRT-1 activator to exert a neuroprotective effect, which may represent a key mechanism underlying its protective effect in EAE. However, since SIRT-1 has both neuroprotective and immune-modulating effects [169,170,171], how much of each property contributes to resveratrol’s protective effect in EAE is still controversial. It was reported by Shindler et al. [158] that the protective effect of SRT501 on EAE was blocked by the administration of the SIRT-1 inhibitor sirtinol and the authors suggested that SIRT-1 activation is a requisite mechanism for the resveratrol’s protective effect on EAE. To further elucidate the role of SIRT-1 in EAE, Nimmagadda et al. directly determined the role of SIRT-1 in regulating EAE by using neuron-specific SIRT-1- transgenic mice that overexpress human SIRT-1 [172]. They found that compared to the wild-type EAE mice, SIRT-1-overexpressed EAE mice showed less severe clinical symptoms and neurological damages (demyelination and axonal injury), decreased pro-inflammatory phenotypes (IFN-γ and IL-17) and an increased anti-inflammatory phenotype (IL-10) in the spinal cord. Nevertheless, it appears that compared with EGCG and curcumin, as discussed above, resveratrol may have a more profound effect in protecting/reversing neurological damages after an inflammatory attack, while it may be less potent in modulating immune and inflammatory responses causing MS/EAE onset. This suggests that the combined use of several phytochemicals may produce additive or synergistic results in preventing and treating MS/EAE.

## 5. The Gut Microbiota: A New Link Between Dietary Phytochemicals and MS Pathogenesis

Recent work implicates the gut microbiota as a key environmental factor influencing disease onset, progression, and therapeutic response. Altered gut microbial composition (dysbiosis) has been associated with skewed peripheral immunity and impaired regulatory capacity in MS patients, with potential effects on intestinal barrier function and the gut–brain axis [173,174,175,176,177,178,179,180,181,182].

The microbiota also critically shapes the fate and bioactivity of dietary phytochemicals. Many polyphenols (for example, EGCG, curcumin, and resveratrol) have low native bioavailability but are extensively transformed by microbial enzymes into metabolites that may have greater stability, absorption, and biological activity [183,184,185,186,187,188,189,190]. In EAE models, such transformations appear functionally important: the administration of a curcumin glucuronide prodrug (CMG) reduced clinical and histological disease and altered microbiota composition in ileal contents and feces, linking site-specific microbial shifts to neuroinflammation outcomes [133].

Together, these data support a dual role for the gut microbiota—as an independent modulator of host immunity and as a biotransformation hub that converts dietary phytochemicals into bioactive molecules with immunomodulatory potential. Phytochemicals may also selectively promote beneficial taxa, creating a bidirectional interaction that could be therapeutically exploited [191,192,193].

For the three phytochemicals reviewed here, there is convincing evidence from animal models to suggest that they would have efficacy in ameliorating EAE symptoms and pathology, which are consistent with, and also further explained by, the favorable modulatory effects of these compounds on immune and inflammatory responses. In addition to immune modulation, an emerging body of evidence suggests that gut microbiota plays a significant role in MS/EAE pathogenesis and response to treatment [176,193,194,195]. Recent studies have shown that the composition of the gut microbiome can influence the onset, progression, and severity of MS and EAE. For example, dysbiosis (an imbalance in gut microbial populations) has been linked to the exacerbation of disease, while a more diverse microbiota seems to confer protective effects, possibly through the modulation of immune responses in the gut-associated lymphoid tissue (GALT) and subsequent effects on systemic immunity.

In summary, the gut microbiota provides a mechanistic link through which dietary phytochemicals may modulate immune and neuroprotective pathways relevant to MS, but further mechanistic and translational research is required.

## 6. Translational Potential and Limitations

Preclinical data for EGCG, curcumin, and resveratrol are promising, but translation to clinical use in MS is challenged by interindividual variability in microbiomes, diet, and genetics. Integrative studies combining metagenomics, metabolomics, and controlled clinical trials are needed to clarify causality, identify actionable microbial–metabolite markers, and guide microbiome-informed stratification or co-interventions in clinical trials.

First, the clinical evidence from human studies is lacking, impeding recommendation for their clinical application as complementary and/or alternative therapies. Given the strong support from animal studies, the potential preventive and therapeutic values of these candidate compounds in MS treatment warrants consideration. However, caution should be practiced when attempting to extrapolate the results from animal models to clinical practice in MS treatment. The issues to consider are the differences present in genetic background, bioavailability and metabolism of these phytochemicals, dose–response pattern, tolerance limit, etc. The most important factor is the differences between the animal EAE and human MS. While EAE is clearly a T cell-mediated autoimmune disease with relatively well-characterized immunopathology to explain the disease induction, the autoimmune hypothesis for MS is still significantly controversial [6], which makes modeling a great challenge. There are a variety of animal models currently used to cover individual aspects and mechanisms relevant in MS pathogenesis, but none can completely produce the pathological and clinical features of MS. This may explain the fact that after clinical trials on a great number of preclinical drugs based on the positive effect in EAE, only a few are proved to be effective and are eventually licensed for clinical use. This situation may as well be expected to be true for the potential clinical application of the naturally occurring phytochemicals which have been proved to be effective in EAE. However, it is worth pointing out that some of these phytochemicals have been shown to improve EAE by favorably affecting multiple aspects of immunological and neurological processes involved in MS/EAE immunopathology, and this may represent a distinct advantage over the conventional drugs, which usually target more specific components in MS pathogenesis. Second, plant-derived phytochemicals are abundant and inexpensive, but poor stability, low water solubility, and low bioavailability limit their ability to reach sufficient levels in both circulation and targeted tissues, e.g., CNS. The development and validation of GMP-grade formulations, adjuvants, or more bioavailable analogs (for example, nanoparticle or liposomal carriers, piperine co-administration, or structural analogs) are necessary to overcome poor oral bioavailability and rapid metabolism and to achieve reproducible therapeutic exposure in plasma and the CNS [196,197]. Several solutions have been tested to resolve this issue, which include the use of adjuvants, structural analogs, and novel delivery systems such as nanoparticles, liposomes, and micelles. To just give a few examples, piperine is used as an adjuvant to enhance the bioavailability of curcumin [198,199,200], EGCG [201], and resveratrol [202]. Structurally modified forms of curcumin (dimethylcurcumin, symmetrical 1, 5-diarylpentadienone, and EF24) are more bioavailable and stable than curcumin [203,204,205]. EGCG analogs have increased stability, bioavailability, and biopotency compared to EGCG [206]. Similarly, the resveratrol analog pterostilbene has higher oral bioavailability and bioactivity than the parent compound [207]. Recently, nanoparticles as a novel delivery system have been used to enhance both the bioavailability and biopotency of polyphenolic compounds, including EGCG, curcumin, and resveratrol [208]. Third, most of the polyphenol compounds used in research are considered safe and they are commercially available as dietary supplements. In animal studies, they are shown to be safe and well-tolerated when used at the doses which can deliver a protective effect to EAE. However, the toxicity and tolerance levels, particularly for long-term use, have not been defined well. It is alarming to notice that in some human studies, EGCG has caused liver toxicity [112,209,210]. Therefore, safety issues should be kept in mind when planning human trials, even though these plant-derived compounds are commonly viewed as harmless. Rigorous safety profiling and the evaluation of potential interactions with approved disease-modifying therapies are necessary [211], given the reports of hepatotoxicity with EGCG and the propensity of phytochemicals to modulate drug-metabolizing enzymes. To mitigate these risks, future trials should perform focused preclinical and clinical safety profiling with hepatic endpoints, use conservative starting doses and stepwise dose escalation, and include routine liver-function monitoring (e.g., ALT, AST, bilirubin). Exclusion criteria should address preexisting liver disease and concomitant hepatotoxic medications/supplements, and all co-medications should be documented. Optimizing formulation and dosing to reduce peak hepatic exposure and assessing potential drug–phytochemical interactions (e.g., CYP, UGT) before trials may further lower risk. Trials should also specify stopping rules and include posttrial follow-up to capture delayed or idiosyncratic events. These measures will help balance potential benefits with patient safety. In addition, accounting for gut microbiota effects on phytochemical metabolism and host responses through microbiome-informed stratification or co-interventions (for example, prebiotics or probiotics) may increase the likelihood of clinical benefit [194,212,213,214]. Furthermore, several primary studies omitted supplier, lot, or purity information for test compounds; where reported, supplier and purity data are noted, but these omissions limit reproducibility and comparability across studies and underscore the need for standardized reporting in future preclinical work.

Finally, interpretation of preclinical EAE data must consider several biases and sources of variability that affect reproducibility and translational relevance. Publication bias toward positive results likely overestimates efficacy in the literature. EAE models differ in immunogen, strain, sex, and induction protocol, and these variables substantially influence disease phenotype and response to interventions; for example, SJL and C57BL/6 mouse models have distinct immunopathologies, and sex differences can alter immune responses and treatment effects. Incomplete reporting of experimental details (including compound purity, dosing metrics, and animal sex) further hampers replication. To improve the evidence base, future studies should preregister protocols where feasible, report methods and negative results fully, include both sexes and multiple strains or models, and apply standardized outcome measures. Recognizing these limitations is essential when extrapolating preclinical phytochemical effects to human MS and in designing robust translational studies.

## 7. Concluding Remarks and Perspectives

In this review, the experimental evidence for the efficacy of EGCG, curcumin, and resveratrol as representative polyphenol phytochemicals for their potential therapeutic value in MS treatment is discussed. It is worth mentioning that protective effect for MS/EAE has been reported for more than a dozen other phytochemicals commonly present in diet. They are not included in this review because the current evidence is insufficient for analysis, and thus, it would be premature to draw conclusions or provide recommendations. These phytochemicals include anthocyanins [215,216,217], apigenin [218], chrysin [219,220], genistein [221,222,223], hesperidin [224,225], myricetin [226,227], naringenin [228], and quercetin [229,230,231].

**Figure 1:**
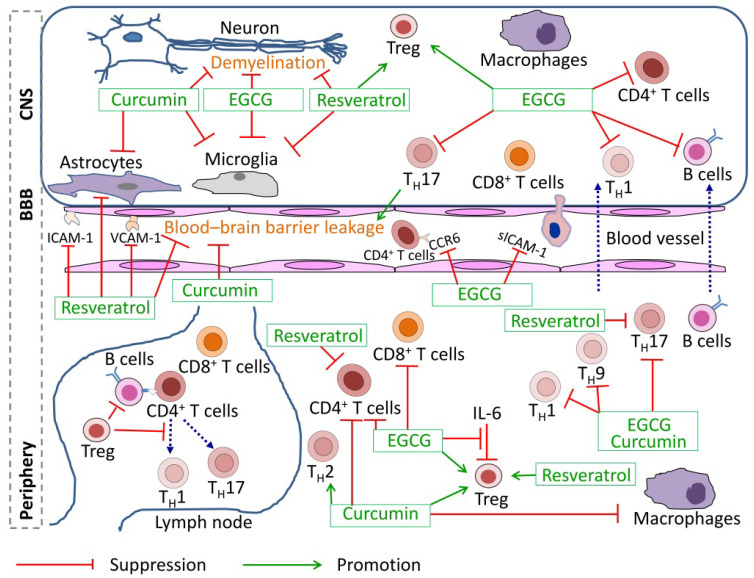
Schematic diagram of the immune-modulatory effects of polyphenols on pathogenesis of MS/EAE. Dietary polyphenols (for example, EGCG, curcumin, and resveratrol) may modulate peripheral immune cells, cross the blood–brain barrier, and act on CNS-resident cells—including immune-competent microglia and immunoreactive astrocytes—thereby reducing neuroinflammation and preventing neuronal demyelination. Abbreviations: BBB: blood–brain barrier; CNS: central nervous system; ICAM-1: intercellular adhesion molecule 1; VCAM-1: vascular cell adhesion molecule 1; CCR6: C–C chemokine receptor type 6; sICAM-1: soluble intercellular adhesion molecule 1; Treg: T regulatory cell.

The components targeted in MS/EAE pathogenesis by these phytochemicals are illustrated in Figure 1, which would help in understanding their working mechanisms. As indicated by the literature review provided above, it is apparent that there is a lot of overlap between these compounds in their effects on various events in MS/EAE pathogenesis. These compounds may modulate gut microbiota composition, which in turn can influence both systemic inflammation and neuroinflammation, further supporting their therapeutic potential. In this sense, phytochemicals not only target immune pathways directly but may also exert indirect effects by promoting a balanced gut microbiome, which could be pivotal in controlling the inflammatory processes in MS/EAE.

It appears that these phytochemicals have some advantageous features that are not shared with the conventional therapies used in treating MS, a complex autoimmune inflammatory disorder. First, these phytochemicals impact multiple aspects of the immune system to differentially modulate varied components in autoimmune inflammation, resulting in a favorable impact on pro- and anti-inflammation balance, instead of narrowly obtaining individual cell types, molecules, and pathways. Second, some of these phytochemicals have neuroprotective effects after immune and inflammatory attacks are fully initiated. This is important because current MS drugs suppress immune and inflammatory episodes but do little to prevent permanent neurological damage, and thus, cannot stop progressive disability. Third, these phytochemicals are relatively safe, easy to obtain, and inexpensive. Therefore, these phytochemicals have the potential to work together with conventional therapies for achieving better efficacy in MS treatment.

The rapid progress in the research for understanding autoimmunity including MS/EAE, together with the increasing availability of emerging novel methodologies, has provided an opportunity to advance the research and clinical practice. Heterogeneity is a well-recognized factor in both the etiology of disease and response to treatment. The rapidly developed “omics” techniques represent a powerful tool to reveal genomic and epigenetic regulations (alleles, single-nucleotide polymorphism, methylation), post-transcriptional regulation (microRNA), and proteomics screening for isomeric forms of protein and post-translational modification. All this information can help identify the intrinsic inter-personal differences and design personalized interventions to achieve optimal outcomes. These advances will help in designing more effective clinical trials to determine the efficacy of these natural compounds in humans against MS.

## Figures and Tables

**Table 1:** Effects of EGCG on animal models of multiple sclerosis.

Animal Model	Sample Size	Intervention	Findings and Conclusions	Ref
Progressive EAE model induced with MOG_35–55_ in 8–10-week-old female C57BL/6 mice	*n* = 17 or 18/group	Oral gavage twice daily from day 12 to day 62 post-immunization. Control: 0.9% NaCl; EGCG: 300 µg EGCG;	EGCG treatment reduced severity of symptom and CNS inflammation, and preserved axons and myelin.	[110]
Progressive EAE model induced with MOG_35–55_ in 6–8-week-old female C57BL/6 mice	*n* = 12/group	Dietary EGCG (0.15%, 0.3%, and 0.6%) from day −30 to 12 (for immune response profiling) or 30 (symptoms and pathology) post-immunization.	EGCG dose-dependently reduced clinical signs and CNS pathology, suppressed antigen-specific T cell proliferation and delayed-type hypersensitivity, increased Treg frequencies, and lowered pro-inflammatory cytokines and Th1/Th17 responses in lymph nodes, spleen, and CNS.	[107]
	*n* = 6/group	Dietary EGCG (0.6%) from day 7 (induction phase) or 12 (effector phase) to day 30 post-immunization.	EGCG delayed onset and attenuated symptoms when started at day 7, and it only attenuated symptoms when started at day 12.	
Relapsing–remitting EAE model induced with PLP_139–151_ in 6–8 week-old female SJL/L mice	*n* = 8/group	Preventive treatment: Oral gavage of EGCG (600 µg/d) started day 9	EGCG delayed disease EAE onset, attenuated disease severity, and reduced CNS inflammatory pathology.	[109]
	*n* = 6/group	Therapeutic treatment: Oral gavage of EGCG (600 µg/d) started when symptom score reached ≥2	EGCG alleviated disability in established EAE.	
Relapsing–remitting EAE model induced with PLP_139–151_ in 6–8 week-old female SJL/L mice	*n* = 6/group	Preventive treatment: Oral gavage of EGCG (600 µg/d) started from day of immunization to day 131.	EGCG reduced disease severity and brain inflammation.	[45]
	*n* = 9/group	Therapeutic treatment: Oral gavage of EGCG (600 µg/d) started from day 12 to 80 post-immunization.	EGCG alleviated clinical symptoms, brain inflammation, and neuronal damage; suppressed lymphocyte proliferation, ROS formation, and TNF-α production; and did not alter IL-4 or IFN-γ levels.	

**Table 2:** Effects of curcumin on animal models of multiple sclerosis.

Animal Model	Sample Size	Intervention	Findings and Conclusions	Ref
Relapsing–remitting EAE induced with guinea pig spinal cord homogenate in Lewis rats	*n* = 8/group	12.5 mg/kg polymerized nano-curcumin (PNC) or curcumin *i.p.* injected daily from day 12–29 post-immunization.	PNC reduced the EAE scores, promoted complete recovery; non-polymerized curcumin did not exert a significant effect.	[72]
		12.5 mg/kg of PNC *i.p.* injected daily from day 0–29 post-immunization.	Prophylactic administration of PNC postponed EAE onset and ameliorated EAE severity	
Progressive EAE induced with MOG_35–55_ in 6–8 week-old female C57BL/6 mice	*n* = 8/group	100 μg curcumin *i.p.* injected every other day from day 0–14 post- immunization.	Curcumin ameliorated EAE, reduced IFN-γ, IL-17, and IL-12 family cytokines in the CNS and lymphoid organs, decreased Th1/Th17 responses, and increased Th2 and Treg responses	[135]
Monophasic EAE induced with MBP_68–86_ in adult female Lewis rats	Control (*n* = 7) 100 mg/kg (*n* = 7) 200 mg/kg (*n* = 23)	Daily oral curcumin (100 or 200 mg/kg) from day 0–14 post- immunization.	Curcumin dose-dependently reduced symptoms, CNS inflammatory cells infiltration, and neural Ag-specific lymphocytes responses.	[137]
Progressive EAE induced with MOG_35–55_ in 4–6 week-old female C57BL/6 mice	Sample size was not specified	100 μg curcumin *i.p.* injected every other day from day 0–14 post-immunization.	Curcumin reduced clinical sore by 60% and T cell proliferation by 41%.	[136]
Relapsing–remitting EAE induced with PLP_139–151_ in 4–6 week-old female SJL/J mice	Sample size was not specified	100 μg curcumin *i.p.* injected every other day from day 0–14 post-immunization.	Curcumin reduced clinical sore by 42% and T cell proliferation by 39%.	
Relapsing–remitting EAE induced with mouse spinal cord homogenate in 4–6 week-old female SJL/J mice (active EAE model)	*n* = 5/group	50 or 100 μg curcumin *i.p.* injected on every other day from day 0–25 post-immunization or adoptive transfer.	Curcumin shortened and lessened symptom severity and reduced CNS inflammation and demyelination in active EAE	[134]
Passive EAE induced by adoptive transfer of encephalitogenic T cells from active EAE mice into naive female recipient SJL/J mice			Curcumin reduced symptom severity in adoptive-transfer EAE, suppressed T cell proliferation, and decreased IFN-γ and IL-12 production by spleen cells, macrophages, and microglia, including IL-12-driven T cell proliferation	

**Table 3:** Effects of resveratrol on animal models of multiple sclerosis.

Animal Model	Sample Size	Intervention	Findings and Conclusions	Ref
Relapsing–remitting EAE induced with MOG_35–55_ in C57BL/6 mice	*n* = 10/group	0, 10, 25, and 50 mg/kg resveratrol *i.p.* injected daily from day 0–20 post-immunization.	Resveratrol reduced symptom severity, preserved BBB integrity, suppressed local inflammation, and inhibited brain NADPH oxidase expression and activity.	[75]
Cuprizone-induced EAE in male C57Bl/6 mice	*n* = 8–10/group	Oral gavage of resveratrol (250 mg/kg) daily from day 0–21 post-immunization.	Resveratrol restored balance and locomotor coordination, reversed demyelination, improved brain mitochondrial function, and reduced oxidative stress and inflammation.	[162]
Relapsing–remitting EAE induced with MOG_35–55_ in 8–12 weeks-old female C57BL/6 mice	*n* = 10/group	30 mg/kg resveratrol *i.p.* injected daily from day 0–7, and mouse bone marrow mesenchymal stem cells (mBM-MSC) *i.v.* injected at day 7 post-immunization.	The combination of resveratrol and mBM-MSCs—unlike either treatment alone—delayed symptom onset, reduced symptom severity, and decreased spinal cord inflammatory infiltration.	[161]
Relapsing–remitting EAE induced with MOG_35–55_ in 6 weeks-old C57BL/6 mice	*n* = 5–6/group	Dietary resveratrol (0.04%, or 20 mg/kg/d) from day −1 to 8 post-immunization (induction phase), from day 14–23 post-immunization (effector phase), or the whole course (day −1 to 63).	All three resveratrol-treated groups showed worse symptoms, and treatment begun during induction yielded significantly higher spinal cord pathology scores than controls.	[163]
Viral model of MS induced with DA strain of TMEV in 5 weeks-old SJL/J mice	*n* = 8/group	Dietary resveratrol (0.04%, or 20 mg/kg/d) from day 35–48 (chronic phase).	Resveratrol treatment produced more severe clinical signs and significantly higher pathology scores than controls.	
Progressive EAE induced with MOG_35–55_ in 6 weeks-old female C57BL/6 mice	*n* = 5/group	Oral gavage of resveratrol (100 mg/kg, 250 mg/kg), or SRT501 (250 mg/kg) daily from day-30 post-immunization.	Both resveratrol and SRT501 (250 mg/kg) delayed EAE onset, prevented neuronal loss, and slowed visual decline, but did not affect CNS inflammation or peripheral/CNS T cells.	[159]
Relapsing–remitting EAE induced with PLP_139–151_ in 6 weeks-old female SJL/J mice	*n* = 3–30/group	Oral gavage of SRT501 (500 mg/kg, 1000 mg/kg) daily from day 8 or 10 to day 14 post-immunization.	SRT501 began before or after optic neuritis onset reduced neuronal damage without altering inflammation, likely via SIRT1 activation.	[158]
Relapsing–remitting EAE induced with PLP_139–151_ in 3 or 8-weeks-old female SJL/J mice (active EAE model)	*n* = 5/group	Dietary resveratrol (0.02%, 0.04%, or 0.08%) from day 0–58 post-immunization.	Resveratrol reduced symptom severity, increased IL-17+IL-10+ T cells and CD4−IFN-γ+ cells in brain and spleen, and suppressed macrophage production of IL-6, IL-12/23p40, IL-12p35, and IL-23p19.	[160]
Passive EAE induced by adoptive transfer of encephalitogenic T cells from active EAE mice into naive female SJL/J recipients	*n* = 5/group	Dietary resveratrol (0.02%, 0.04%, or 0.08%) from day −7 to day 20 post-immunization.	Resveratrol-pretreated recipient mice had reduced severity of symptoms after adoptive transfer.	
Progressive EAE induced with MOG_35–55_ in 6–8 weeks-old C57BL/6 mice	*n* = 6/group	Oral gavage of resveratrol (100 mg/kg, 250 mg/kg) daily oral gavage from day 2–30 post-immunization.	Resveratrol delayed disease onset, lessened symptoms, and reduced inflammation in the spinal cord and peripheral blood.	[157]
Relapsing–remitting EAE induced with PLP_139–151_ in 6 weeks-old SJL/J mice	*n* = 15 eye	Intravitreal injection of 13 μM SRT501 on days 0, 3, 7, and 11 after immunization, and mice euthanized on day 14	SRT501 increased retinal ganglion cell survival during acute optic neuritis, likely via SIRT1 activation.	[156]

## Data Availability

No new data were generated or analyzed in this study. This article is based solely on previously published research.

## Abbreviations

The following abbreviations are used in this manuscript:

MS	multiple sclerosis
EGCG	epigallocatechin-3-gallate
CNS	central nervous system
EAE	experimental autoimmune encephalomyelitis
BMP	basic myelin protein
PLP	proteolipid protein
MOG	myelin oligodendrocyte glycoprotein
BBB	blood–brain barrier
Treg	regulatory T cells
APC	antigen-presenting cells
ROS	reactive oxygen species
RGC	retinal ganglion cells
EGC	epigallocatechin
sICAM-1	soluble intercellular adhesion molecule-1
TLR	toll-like receptors
